# Effects of macronutrient intake on the lifespan and fecundity of the marula fruit fly, *Ceratitis cosyra* (Tephritidae): Extreme lifespan in a host specialist

**DOI:** 10.1002/ece3.3543

**Published:** 2017-10-22

**Authors:** Kevin Malod, C. Ruth Archer, John Hunt, Susan W. Nicolson, Christopher W. Weldon

**Affiliations:** ^1^ Department of Zoology and Entomology University of Pretoria Hatfield South Africa; ^2^ Centre for Ecology and Conservation College of Life and Environmental Sciences University of Exeter Cornwall UK; ^3^ School of Science and Health Western Sydney University Penrith NSW Australia

**Keywords:** host specialization, life‐history strategy, lifespan, nutritional geometry, tephritidae, trade‐off

## Abstract

In insects, lifespan and reproduction are strongly associated with nutrition. The ratio and amount of nutrients individuals consume affect their life expectancy and reproductive investment. The geometric framework (GF) enables us to explore how animals regulate their intake of multiple nutrients simultaneously and determine how these nutrients interact to affect life‐history traits of interest. Studies using the GF on host‐generalist tephritid flies have highlighted trade‐offs between longevity and reproductive effort in females, mediated by the protein‐to‐carbohydrate (P:C) ratio that individuals consume. Here, we tested how P and C intake affect lifespan (LS) in both sexes, and female lifetime (LEP), and daily (DEP) egg production, in *Ceratitis cosyra*, a host‐specialist tephritid fly. We then determined the P:C ratio that *C. cosyra* defends when offered a choice of foods. Female LS was optimized at a 0:1 P:C ratio, whereas to maximize their fecundity, females needed to consume a higher P:C ratio (LEP = 1:6 P:C; DEP = 1:2.5 P:C). In males, LS was also optimized at a low P:C ratio of 1:10. However, when given the opportunity to regulate their intake, both sexes actively defended a 1:3 P:C ratio, which is closer to the target for DEP than either LS or LEP. Our results show that female *C. cosyra* experienced a moderate trade‐off between LS and fecundity. Moreover, the diets that maximized expression of LEP and DEP were of lower P:C ratio than those required for optimal expression of these traits in host‐generalist tephritids or other generalist insects.

## 1 INTRODUCTION

The amount of food ingested by an individual, and the ratio of specific nutrients contained in that food, is critical to its fitness (Simpson & Raubenheimer, 2012). Expression of life‐history traits such as growth (Dussutour, Latty, Beekman, & Simpson, 2010), reproduction, and survival (Jensen, Mcclure, Priest, & Hunt, 2015) all depend on nutrition. The relationship between the ratio of nutrients consumed and life‐history traits can be modeled using the “Geometric Framework” (GF; Simpson & Raubenheimer, 2012). This approach determines how intake of multiple nutrients (usually two nutrients, such as protein and carbohydrate, but the GF is not limited to these two dimensions) and energy (calories) interact to affect multiple traits of interest. The GF has been instrumental in challenging the notion that lifespan is extended when animals experience caloric restriction (Lee et al., 2008) and has shown that both the ratio and total amount of nutrients that individuals eat affect their survival (Chen, Wei, Wei, & Yuan, 2013; Le Couteur et al., 2016; Lee et al., 2008). In general, diets promoting extended lifespan in herbivorous insects are usually of low P:C ratio, whereas high P:C ratios often severely reduce survival (Behmer, 2009; Le Couteur et al., 2016; Le Gall & Behmer, 2014).

The GF also shows that in many species, maximizing survival and reproductive effort requires eating different ratios of nutrients. For example, female *Drosophila melanogaster* needs to consume high P:C ratio diets to maximize fertility, but high protein intake reduces female survival (Jensen et al., 2015). In this situation, females cannot maximize fertility and lifespan at the same intake of nutrients, and so they face a trade‐off between these important life‐history traits. Trade‐offs between reproduction and lifespan are common: in general, investing in reproduction is costly and usually shortens lifespan (Flatt, 2011) and accordingly reproductive effort in short‐lived species is typically intense and early in life. Conversely, long‐lived species usually exhibit weak early reproductive effort (Hayward, Nenko, & Lummaa, 2015; Kirkwood & Rose, 1991). However, the GF shows us that particular nutrients can mediate trade‐offs and the severity of trade‐offs can differ between the sexes. For example, dietary proteins are detrimental to lifespan (Fanson, Fanson, & Taylor, 2012), but unlike males, females require higher P:C ratios to maximize reproductive effort (e.g., egg production; Le Couteur et al., 2016). Thus, the trade‐off between lifespan and reproduction seems to be more important in females than in males, although there are data showing that this trade‐off also occurs in male Mexican fruit flies *Anastrepha ludens*, and is related to dietary protein (Harwood et al., 2015).

Dietary optima and trade‐offs might also differ at the interspecific level between closely related species that differ in their habitat use. Two evolutionary strategies can be distinguished with regard to habitat use: specialization and generalization (Jaenike, 1990). When applied to diet selection and consumption, a specialist species refers to one that utilizes a narrow range of food resources. Diet specialization is prevalent among herbivorous insects (Cunningham, Carlsson, Villa, Dekker, & Clarke, 2016). Generalist herbivore species may also be referred to as polyphagous (feed on plants from a wide range of plant families), and specialists may be classed as oligophagous (feed on few species but usually within a single plant family) or monophagous (feed on a single plant species). In addition to differences in the range of foods in their diet, specialist and generalist species also differ in their nutrient regulation strategies. In the GF approach, the ratio and amount of nutrients that should be ingested to optimize a particular life‐history trait are referred to as the intake target (Raubenheimer, Simpson, & Mayntz, 2009).

To date, studies using a GF approach have mainly focused on generalist species. The true fruit flies (Diptera: Tephritidae) are a strong model to study how dietary niche breadth modulates nutrient regulation and the relationship between nutrition and fitness traits. The Tephritidae include several species of highly invasive, polyphagous pests of fruit production (Lux, Ekesi, Dimbi, Mohamed, & Billah, 2003; Malacrida et al., 2007). Generalist species such as the Mediterranean fruit fly *Ceratitis capitata*, or the oriental fruit fly *Bactrocera dorsalis*, as well as specialist species, such as the olive fly *Bactrocera oleae*, can be invasive and of economic importance (Daane & Johnson, 2010; Ekesi, De Meyer, Mohamed, Virgilio, & Borgemeister, 2016; Malacrida et al., 2007). In the wild, adult tephritids have been recorded acquiring their nutrients from different food sources: fruit juice, nectar, pollen, extrafloral glandular secretions, honeydew, bird feces, and bacteria (Drew, Courtice, & Teakle, 1983; Hendrichs, Lauzon, Cooley, & Prokopy, 1993; Manrakhan & Lux, 2006). Due to their economic impact on fruit trade, the link between nutrition, reproduction, and lifespan has been relatively well studied in generalist tephritids using dietary manipulations, where hydrolyzed yeast was used as a source of protein (Carey et al., 2008; Fanson, Weldon, Perez‐Staples, Simpson, & Taylor, 2009; Harwood et al., 2013; Liedo, Carey, Ingram, & Zou, 2012; Oviedo et al., 2011). However, as hydrolyzed yeast is a mixture of macro‐ and micronutrients, it is preferable to use protein only (a mixture of amino acids) to ensure that the observed effect can be attributed to the macronutrient and not to the quality of the industrial yeast (Fanson & Taylor, 2012; Piper & Partridge, 2007). To date, the GF has been used to specifically relate macronutrient intake and the lifespan–reproduction trade‐off in only one tephritid fly *Bactrocera tryoni*, which is a generalist species (Fanson & Taylor, 2012).

Here, we aim to unravel how the lifespan–reproduction trade‐off is modulated by nutrient intake in a tephritid species with a more restricted host range than those that have been studied previously and identify which nutrient regulation strategy this species adopts. To do so, we combined the GF approach with an advanced statistical method recently developed for GF studies that enables us to evaluate the strength of trade‐offs that may occur between fitness traits (Bunning et al., 2015, 2016; Rapkin et al., 2015). In this study, we apply the GF to a tephritid fly that is more specialized, the marula fruit fly *Ceratitis cosyra*, which is indigenous to Africa and a serious pest for mango production (Biber‐Freudenberg, Ziemacki, Tonnang, & Borgemeister, 2016; Lux et al., 2003). In a strict sense, *C. cosyra* may be regarded as polyphagous because it has been recorded to infest fruit of 28 identified plant species from 16 families (De Meyer et al., 2002). However, its geographic distribution closely follows that of the marula tree *Sclerocarya birrea* (De Villiers, Manrakhan, Addison, & Hattingh, 2013), and females prefer to oviposit in the fruits of marula and mango *Mangifera indica*, both of which are in the family Anacardiaceae. Consequently, *C. cosyra* has been considered as an oligophagous species by some authors (Manrakhan & Lux, 2006). As a major pest of mango production, the distribution, phenology, and host preferences of *C. cosyra* have been relatively well studied. However, this is not the case for its life‐history traits, and to date, only one study has focused on the effects of nutrition on lifespan and reproduction in *C. cosyra* (Manrakhan & Lux, 2006). As females oviposit in the fruit of few plant species, host availability may render their reproductive output sensitive to seasonal variation. Therefore, it may be that *C osyra* will exhibit a longer lifespan than more generalist tephritid species to cope with the seasonal availability of fruit. Similarly, it may be expected that *C. cosyra* will follow a nutrient regulation strategy that promotes extended lifetime egg production and a longer lifespan, that is, regulation of intake toward a lower P:C ratio. This represents a first step toward understanding the diversity of life‐history and nutrient regulation strategies in tephritid flies with regard to their patterns of host use.

## 2 MATERIALS AND METHODS

### 2.1 Fly husbandry

Pupae of the marula fruit fly, *C. cosyra*, were collected from infested mangoes from various locations in Mpumalanga and Limpopo provinces, South Africa. Offspring of these flies constituted our fly stock, maintained in a ~23°C, 14:10 dark light photoperiod climate room. Adults were kept in groups of approximatively 200 flies in 5‐L plastic cages with unrestricted access to food (hydrolyzed yeast and sugar in separate dishes) and water (water‐soaked cotton wool). Experimental flies were obtained by allowing females from stock to lay eggs on a 125‐ml plastic container (Plastilon, South Africa) covered with a layer of laboratory film (Parafilm M, Bemis, USA) pierced several times and in which was placed a guava juice‐soaked tissue paper. Eggs were then harvested and transferred into a 125‐ml plastic container filled with artificial carrot‐based diet (Citrus Research International, Nelspruit, South Africa). The container was then placed in a 1‐L plastic box with a layer of sand and a ventilated lid. Once the pupal phase was reached (15 days at 25°C), the sand was sifted and the retrieved pupae placed in a Petri dish (ø 65 mm) and transferred into a 5‐L plastic cage with only water available until adult emergence.

### 2.2 Experimental diets and consumption

Eighteen diets (Table S1) were prepared that varied in their P:C ratio (0:1; 1:8; 1:4; 1:2; 1:1; 2:1) and total concentration of P + C (45, 180, and 360 g/L) as in Jensen et al. (2015). Sucrose was used as a source of carbohydrate (C), and a blend of 18 free amino acids (Table S2) was used as a source of protein (P). Each diet also contained equal concentrations of micronutrients (Table S1). Either one (no‐choice experiment) or two (choice experiment) experimental diets were provided to individual flies on their day of emergence in 200‐μl pipette tips (ROLL s.a.s, Italy), capped loosely with putty‐like adhesive (Bostik, South Africa).

The volume of food consumed was determined by measuring pipette tips containing liquid diets with 1‐mm scale graph paper (Canson, France). We replaced pipette tips every 4 days or earlier if running low. Food was measured when provided to a fly and 2 days later and then again when food was replaced. The amount of ingested liquid was calculated from the difference between the initial length (for 100 μl) and the remaining length of diet in the pipette. Linear measurements of consumption were converted into volumes with a mathematical function that was obtained from a standard curve (Fig. S1). In the no‐choice experiment, each diet had three pipette tips used as controls (i.e., placed in the climate room, but in containers without flies) to assess the evaporation rate. In the choice experiment, two containers with two pipette tips per diet were maintained in the climate room to measure evaporation rate. The amount of volume lost was then used to correct consumption for evaporation.

### 2.3 Experiment I: no‐choice of diet on five nutritional rails at three concentrations

To analyze the effect of P and C on lifespan (LS) and reproduction, each of the 18 experimental diets was provided separately to seven females and seven males, (*n* = 288 flies, Table S3). Within 24 hr of emergence, virgin females and males were placed individually in containers (125 ml). Each container was supplied with two 200‐μl pipette tips, one containing filtered water and one containing 100 μl of experimental diet. Mortality was checked daily. We measured reproductive effort by recording fecundity. We placed an ovipositing Petri dish, filled with 2.5 ml of 10% orange essence solution (Robertsons, Johannesburg, South Africa) that covered the entire base of the container in cages containing females (Fig. S2). Eggs were counted every 4 days when dishes were replaced. These data allowed us to estimate daily egg production (eggs/day, DEP) for an individual, and lifetime egg production (i.e., giving number of eggs laid throughout entire lifespan, LEP). The average temperature and relative humidity (RH) in the CR during the no‐choice experiment were 23.2 ± 2.5°C and 47% ± 9%.

### 2.4 Experiment II: nutrient intake under dietary choice

To assess the regulation of nutrient intake in male and female *C. cosyra*, consumption of carbohydrate and protein was recorded when flies were allowed dietary choice using established methods (Jensen et al., 2015; Maklakov et al., 2008). Flies were maintained as in the no‐choice experiment, the only difference being that instead of one diet, they were given a pair of diets. Flies were randomly assigned to one of the following dietary pairs (Fig. S3): Pair 1: 1:1 (180 g/L) vs. 0:1 (180 g/L); Pair 2: 1:1 (180 g/L) vs. 0:1 (360 g/L); Pair 3: 1:1 (360 g/L) vs. 0:1 (180 g/L); Pair 4: 1:1 (360 g/L) vs. 0:1 (360 g/L); Pair 5: 1:2 (360 g/L) vs. 0:1 (360 g/L). For each diet in the pair, 100 μl was provided in a different pipette tip. The diet pairs were tested on eight flies of each sex, in two blocks (*n*_total_ = 160 flies). Diet consumption was recorded every 2 days, starting within 24 hr after emergence over a period of 16 days. Flies dying before the end of the experiment or escaping were removed from data analysis. The average temperature and RH during the first replicate were 21.5 ± 3°C and 52% ± 5%, and during the second replicate 21.6 ± 2.9°C and 54% ± 3%.

### 2.5 Statistical analyses

In Experiment I, as longevity among the diet groups was highly variable, we divided total consumption by days lived to express male and female consumption in mg per day so that consumption by individuals was more comparable. Moreover, as the data distribution was skewed, we used a log10(× + 1) transformation on nutrient intake and traits (LS, LEP, and DEP). Data from flies escaping during the experiment or dying from non‐natural death (trapped in a drop of liquid diet) were removed. We then followed the procedure described in detail in Rapkin et al. (2015) and Bunning et al. (2016). In brief, we used a multivariate response surface approach to estimate the linear and nonlinear (interactions between P × P, C × C, and P × C) effects of P and C on response variables (LS, LEP, DEP) for each sex. A sequential approach was used to compare nutritional landscapes across sexes for LS and across the different traits in females (LS, LEP, and DEP). When an overall significant difference was detected a univariate analysis was used to determine which nutrient contributed to the effect. To determine if the variable responses were optimized in the same region of the nutrient space, we calculated the angle (θ) between the linear vectors for the two traits being compared using trigonometry and the 95% confidence interval for θ as explained in Rapkin et al. (2015). To visualize the data, nutritional landscapes were constructed with the function Tps from the package FIELDS in R Software (R core team, version 3.3.1), and raw data were used to construct the surface responses.

In Experiment II, the intake of nutrients expected if individuals fed at random from each diet was calculated for each fly. These expected intakes were subtracted from the observed intake of nutrients, and the difference was compared with zero (one sample t test). If values were not significantly different, this indicated that flies ate each diet randomly. We then compared intake from each diet pair with a t test to determine the preferred diet for females and males. A multivariate analysis of variance (MANOVA) was run to determine how nutrient intake differed across diet pairs, sexes, and replicates. We included sex and diet pair as fixed effects and replicate as a random effect plus all interactions between sex, diet pair, and replicate. The response variables in this analysis were the total intake of P and C. We here expressed total intakes in mg rather than in mg per day as all flies were subjected to the dietary choice experiment over the same duration. Univariate ANOVAs were used on significant effects to determine which nutrient contributed to the significant effects found in the MANOVA. As diet pair was the only significant fixed effect, we used Bonferroni post hoc tests to determine which diet pairs were different. For each sex and each diet pair, we calculated a cumulative intake for P and C, then a regulated intake point as the mean total intake of P and C across all diet pairs.

## 3 RESULTS

### 3.1 Experiment I: no‐choice on five nutritional rails at three concentrations

**Figure 1:**
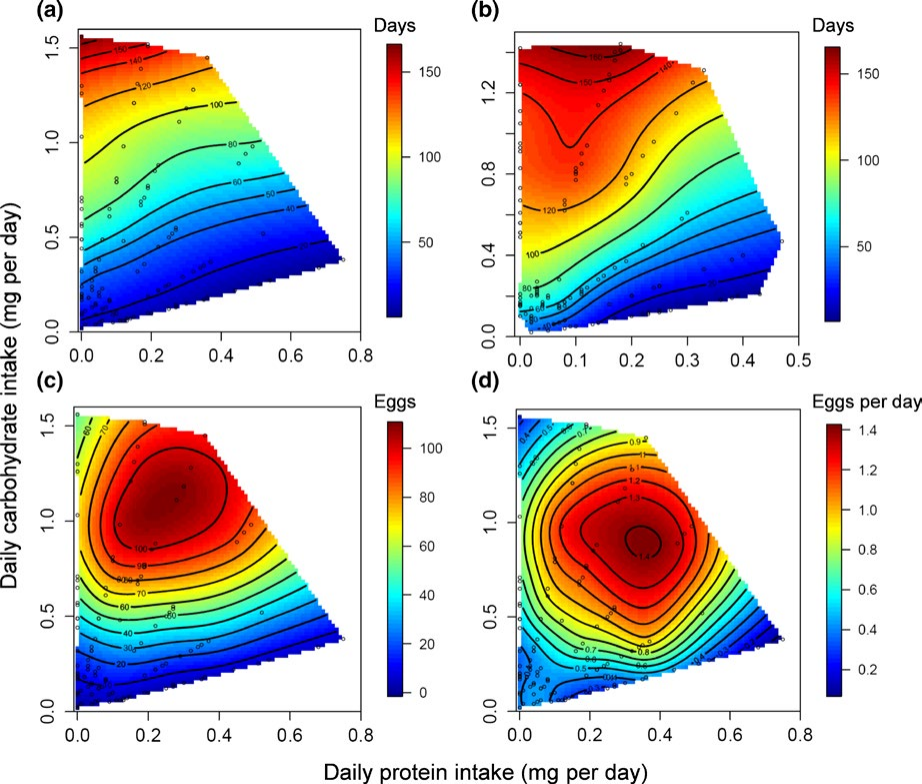
Nutritional landscapes for female lifespan (a), male lifespan (b), female lifetime reproductive effort (c), and female daily reproductive effort (d). Female reproductive effort was measured through egg production. The color gradient ranging from red to blue indicates how individuals perform for a trait on a specific P:C intake (moving toward red shows values for a trait are increasing). For female nutritional landscapes *n* = 119, for male *n* = 132

In both sexes, individuals lived longest when eating a low P:C ratio (Fig. S4). In females, intake of P and C had a significant linear effect on LS (Table 1), with LS decreasing with intake of P and increasing with intake of C (Fig. 1). The quadratic effects of C had a significant negative effect on female LS, due to a peak in expression in females fed a high carbohydrate diet. LS peaked at a 0:1 P:C ratio. No significant correlation effects were found in females. In males, both nutrients had a significant linear effect on LS (Table 1); as in females, LS decreased with intake of P and increased with intake of C. We found a significant quadratic effect only for C, indicating that LS was optimized at a low P:C ratio in the nutrient space. A significant positive correlational effect was found, indicating a peak in the nutritional landscape at a high intake of both nutrients. LS peaked at a 1:10 P:C ratio.

**Table 1:** The effects of P and C intake on lifespan (LS) in males and females and on daily egg production (DEP) and lifetime egg production (LEP)

Response variables	Linear effects		Nonlinear effects		
	P	C	P × P	C × C	P × C
Males					
Lifespan					
Coefficient ± *SE*	−0.41 ± 0.06	0.67 ± 0.06	0.02 ± 0.05	−0.31 ± 0.07	0.28 ± 0.06
*t*_131_	7.08	11.53	0.38	4.59	4.72
*p* value	**0.0001**	**0.0001**	0.70	**0.0001**	**0.0001**
Females					
Lifespan					
Coefficient ± *SE*	−0.25 ± 0.06	0.79 ± 0.06	−0.04 ± 0.05	−0.13 ± 0.06	0.06 ± 0.07
*t*_118_	4.32	16.53	0.95	2.12	0.94
*p* value	**0.0001**	**0.0001**	0.34	**0.04**	0.35
Daily egg production					
Coefficient ± *SE*	0.01 ± 0.09	0.32 ± 0.09	−0.15 ± 0.07	−0.24 ± 0.09	0.23 ± 0.10
*t*_118_	0.12	3.61	2.26	2.57	2.21
*p* value	0.91	**0.0001**	**0.03**	**0.01**	**0.03**
Lifetime egg production					
Coefficient ± *SE*	−0.14 ± 0.07	0.65 ± 0.07	−0.11 ± 0.05	−0.25 ± 0.07	0.25 ± 0.08
*t*_118_	1.94	9.00	2.06	3.39	3.09
*p* value	0.06	**0.0001**	**0.04**	**0.001**	**0.003**

Values in bold indicate significant effects with *p* < 0.05.

When female and male nutritional landscapes were compared with a sequential building approach (Table 2), we found differences in the significant linear effect of P on LS. This is because P had a more negative impact on males than females (Table 1). However, while the magnitude of the nutritional gradient differed between female and male LS, the angle difference between the linear vectors was small (Table 2), indicating that female and male LS peaked in a very similar region of the nutritional landscape.

**Table 2:** Comparison of male and female nutritional landscapes for lifespan (LS), and LS, daily egg production (DEP), and lifetime egg production (LEP) in females

	SS_*R*_	SS_*C*_	DF_1_	DF_2_	*F*	*p* value	θ	95% CI
LS: Female vs. Male								
Linear	103.87	100.05	2	245	3.42	**0.01** ^**A**^	13.98°	3.17°, 25.23°
Quadratic	95.47	94.51	2	241	1.22	0.30		
Correlational	88.54	86.46	1	239	5.73	**0.02**		
LS vs. DEP								
Linear	165.07	150.54	2	232	11.20	**0.0001** ^**B**^	21.98°	0.00°, 49.37°
Quadratic	142.70	141.38	2	228	1.06	0.35		
Correlational	138.02	136.98	1	226	1.71	0.19		
LS vs. LEP								
Linear	115.91	114.35	2	232	1.58	0.21	7.28°	0.00°, 17.51°
Quadratic	107.48	106.78	2	228	0.75	0.47		
Correlational	102.85	101.49	1	226	3.03	0.08		
DEP vs. LEP								
Linear	181.83	175.11	2	232	4.45	**0.01** ^**C**^	18.45°	0.00°, 45.23°
Quadratic	161.93	161.75	2	228	0.13	0.88		
Correlational	152.75	152.73	1	226	0.03	0.86		

Univariate tests: ^A^ P: *F*_1,245_ = 3.89, *p *=* *.05; C: *F*_1,245_ = 2.27, *p *=* *.13; ^B^ P: *F*_1,232_ = 6.04, *p *=* *.015; C: *F*_1,232_ = 19.40, *p *=* *.0001; ^C^P: *F*_1,232_ = 1.73, *p *=* *.19; C: *F*_1,232_ = 8.21, *p *=* *.005. Values in bold indicate significant effects with *p* < 0.05.

For reproductive traits, only C intake had a significant linear effect on DEP and LEP (Table 1), with both traits increasing with the intake of C. The quadratic effects for P and C were significant for both reproductive traits, indicating that there was a peak in the nutrient landscape. A peak was detected for DEP at approximatively 1:2.5, whereas LEP peaked at a 1:6 P:C ratio. A significant positive correlational effect was found in both traits, indicating an increase in the trait values with increased intake of both nutrients.

When we compared female LS to reproductive traits, we only found linear differences between LS and DEP (Table 2) and univariate tests revealed that this effect was driven by both nutrients. This is because P negatively impacted LS but not DEP where no significant linear effect was found. Also, the positive effect of C was more important for LS than DEP. The moderate angle between LS and DEP indicated that these traits peaked in slightly different regions of the nutritional landscape. We found no difference between LS and LEP, and the angle between traits was very small. Comparison of the nutritional landscapes between reproductive traits revealed a significant linear effect only between DEP and LEP, and this was driven by C intake only (Table 2). The angle was moderate and indicates that DEP and LEP peaked in slightly different regions of the nutritional landscape.

### 3.2 Experiment II: nutrient intake under dietary choice

**Figure 2:**
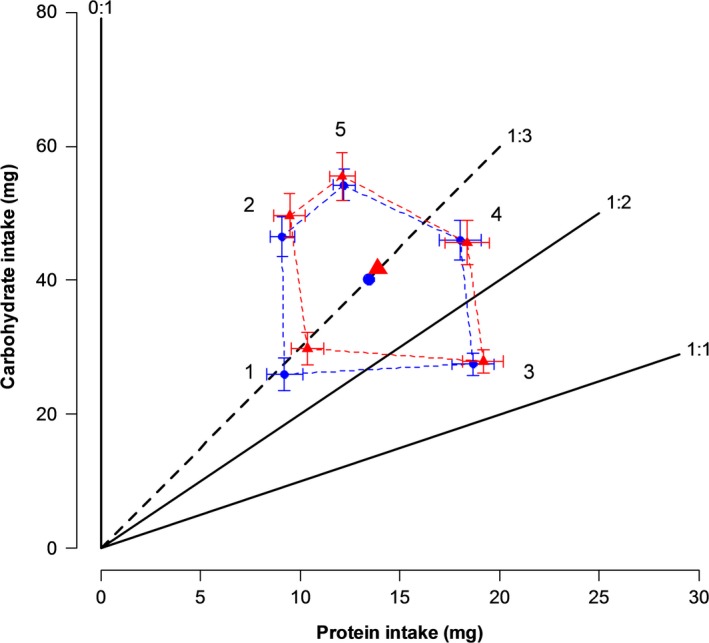
Average total intake (±*SE*) of P and C when females and males were given the choice between two diets over a 16‐day feeding period. Females are represented by red triangles and males by blue circles. The regulated intake points are represented by a large red triangle for females and a large blue circle for males, and both are located on the 1:3 nutritional rail. Diet pairs are represented by numbers: Pair 1: 1:1 (180 g/L) vs. 0:1 (180 g/L); Pair 2: 1:1 (180 g/L) vs. 0:1 (360 g/L); Pair 3: 1:1 (360 g/L) vs. 0:1 (180 g/L); Pair 4: 1:1 (360 g/L) vs. 0:1 (360 g/L); Pair 5: 1:2 (360 g/L) vs. 0:1 (360 g/L). For each sex and pair *n* = 16

When we compared the difference between expected intake and observed intake of P and C to a mean of zero, we found that females and males were not consuming nutrients randomly and so were regulating their intake of each nutrient. We observed a significant preference for P across diet pairs in both sexes, except in pair 3 (1:1 (360 g/L) vs. 0:1 (180 g/L)) where flies were feeding at random (Fig. S5). Both sexes showed a significant preference within the pairs for the diet containing P (Fig. S6); this was not observed once again in pair 3 where no preference was detected. The cumulative intake for P and C for each pair was similar between the sexes. The regulated intake points calculated for both sexes were very similar (female: P = 13.9 mg, C = 41.7 mg; male: P = 13.4 mg, C = 40 mg), and both located on the nutritional rail representing a 1:3 P:C ratio (Fig. 2).

Multivariate analysis of variance showed that replicate and pair were the only two terms affecting P and C intake, and their interaction (Replicate × Pair) also impacted P intake (Table 3). We counted replicate as a random effect because the effects of this term were due to variation in diet evaporation (Table S4). This led to a slightly smaller quantity of nutrients consumed when we corrected total consumption with evaporation in the first replicate. Thus, we did not take account of replicates and only performed univariate ANOVAs for the factor pair, the only term having a significant effect. Areas defended by females and males are displayed separately for each replicate in the supplementary material section (Figs. S7–S8). A significant effect of pair on P and C intake was detected by univariate ANOVA (Table 3), and Bonferroni post hoc tests were used to identify pairs that differed from one another. Post hoc tests indicate that the intake of P was not different between pairs 1 and 2, pairs 3 and 4, whereas pair 5 was significantly different from the other pairs. The intake of C did not differ between pairs 2 and 3, but pairs 1, 4, and 5 were significantly different from each other.

**Table 3:** Differences of P and C total intake (mg) between sexes, diet pairs, and replicates. Univariate ANOVAs are provided only for the relevant term having a significant effect in the multivariate model

	MANOVA			
	Nutrient	*F*	*df*	*p* value
Replicate	P	20.78	1	**0.009**
	C	63.65	1	**0.024**
Sex	P	0.48	1	0.612
	C	0.35	1	0.659
Pair	P	38.38	4	**0.002** ^**A**^
	C	41.63	4	**0.001** ^**B**^
Replicate × Sex	P	3.05	1	0.155
	C	3.70	1	0.126
Replicate × Pair	P	10.07	4	**0.02**
	C	2.41	4	0.208
Sex × Pair	P	0.58	4	0.696
	C	0.29	4	0.872
Replicate × Sex × Pair	P	1.17	4	0.325
	C	2.08	4	0.087

Univariate ANOVAs: ^A^P: *F* = 139.14, *df* = 4, *p* value <.001; ^B^C: *F* = 38.76, *df* = 4, *p* value <.001. Values in bold indicate significant effects with *p* < 0.05.

## 4 DISCUSSION

We aimed to determine how the amount and ratio of protein and carbohydrate ingested affect lifespan (LS) and reproductive effort (LEP and DEP) in a fruit fly species with a limited host range. Regardless of sex, both nutrients had a clear effect on lifespan: increasing the P:C ratio reduced lifespan, while decreasing this ratio improved lifespan. Thus, we found that female and male lifespans were maximized at a high intake of both nutrients and low P:C ratios, 0:1 and 1:10, respectively. Surprisingly, carbohydrate intake had a greater effect on female reproductive effort than protein intake, and accordingly, LEP and DEP were maximized at a low‐to‐moderate P:C ratio of 1:6 (LEP) and 1:2.5 (DEP). In addition, reproductive effort was optimized at a lower intake of both nutrients in comparison with lifespan. When comparing nutritional landscapes, we found a small difference between female and male lifespan, which was driven by the intake of protein. Indeed, females performed very well without ingesting any protein, whereas male lifespan required an intake of protein to be optimized. This led to males consuming a slightly higher P:C ratio than females. Our results show that female lifespan and LEP are optimized in a very similar region of the nutrient space. However, the analysis revealed moderate trade‐offs between lifespan and DEP, as well as between LEP and DEP. In other words, long‐lived females could also produce many eggs over their entire lifetime, but to maximize DEP required consumption of higher P:C ratios and this was associated with reduced survival. In a dietary choice situation, both sexes defended a 1:3 P:C ratio, which would promote strong DEP and a fairly good LEP in females, but lifespan in both sexes would be moderate compared with lower P:C ratios. Thus, *C. cosyra* females opted for a protein and carbohydrate intake promoting strong egg production per day, rather than across their entire lives. The effects of protein and carbohydrate intake on lifespan in our study species were similar to those observed in other studies applying the GF to Tephritidae (Fanson & Taylor, 2012; Fanson et al., 2009), Diptera (Jensen et al., 2015; Lee et al., 2008), and other herbivorous insect orders (Harrison, Raubenheimer, Simpson, Godin, & Bertram, 2014; Maklakov et al., 2008). As in these other studies, we found that lifespan was optimized when flies consumed high quantities of a carbohydrate‐biased diet.

We show here that in female *C. cosyra*, both macronutrients are important for reproductive effort, but a more protein‐biased intake is required to optimize reproductive traits than for lifespan. However, compared with other species, optima for LEP and DEP were less protein biased in *C. cosyra*. For example, LEP and DEP were optimized at a 1:4 and 1:1 P:C ratio in *B. tryoni* (Fanson & Taylor, 2012), and 1:4 (or 1:2 in Jensen et al., 2015) and 1:2 in *D. melanogaster* (Jensen et al., 2015; Lee et al., 2008). In addition, even when optimized, DEP in *C. cosyra* was far lower than in the generalist species *B. tryoni*, while LEP was similar between unmated females of the two species (Fanson et al., 2009). Females were able to produce a considerable number of eggs over their lifetime, even on low P:C ratios, but at a very low rate (i.e., egg production spread over lifespan). If a long life improves female fitness by allowing greater egg production, then there may be strong selection on female lifespan in this species, explaining why female *C. cosyra* live for longer than most other tephritids. This could explain why carbohydrate is the important nutrient for female lifetime reproductive effort, as high carbohydrate intake at a low P:C ratio promotes survival, and this positively correlates with lifetime reproductive success. Our results show that there were peaks in the nutritional landscape for LEP and DEP as a function of protein intake, and the nutrient blend for these traits (in particular DEP) is more protein biased than the optimum for lifespan. Clearly, protein is important for producing eggs. This mirrors findings in other species, as dietary protein is essential for female egg laying in generalist and specialist tephritids (Harwood et al., 2013) as well as in other insects including crickets and *Drosophila* (Jensen et al., 2015; Maklakov et al., 2008).

Using the GF approach, similar results were found in *Nauphoeta cinerea*, where females had a low protein requirement for reproduction (Bunning et al., 2016). Interestingly, *N. cinerea* is also known to have a particularly long lifespan of up to 3 years in the laboratory (Moore & Moore, 2001). Bunning et al. (2016) suggested that the low protein requirement for reproduction in *N. cinerea* females was potentially due to their capacity to store excess protein as nitrogen through endosymbiotic interactions. Hence, if females had access to a protein source as juveniles, it might have been possible for them to restore this protein stock when fed on low P:C diet in their adult stage. Similar mechanisms are present in Tephritidae: the microbiota in the gut of *B. oleae* transforms nitrogen into amino acids essential for protein synthesis (Ben‐Yozef, Pasternak, Jurkevitch, & Yuval, 2014). Moreover, the larval diet of our experimental flies contained protein. The fact that adult females fed on carbohydrate only were still able to lay eggs suggests that protein may have been stored from the larval stage in a form that can be retrieved by adults. Storing of protein from the larval stage has been observed in *C. capitata* where protein content measured in emerging adults corresponded with the protein content in pupating larvae (Nestel, Nemny‐Lavy, & Chang, 2004).

Both sexes regulated their nutrient intake at a higher P:C ratio (1:3) than we predicted for a species with a more limited host range where a long life is important for surviving when the host is limiting. Based on the results of the no‐choice experiment, the nutrient regulation strategy adopted by *C. cosyra* is clearly promoting female reproductive effort, and more particularly DEP. While expression of these fitness traits was maximized in *C. cosyra* at lower P:C ratios than in *B. tryoni*, our results in the dietary choice experiment are similar to those obtained for *B. tryoni* (Fanson et al., 2009), with both species defending a 1:3 ratio. The regulated P:C ratio of *B. tryoni* is less protein biased than its optima for DEP and LEP but has a higher ratio than the optimum for lifespan. In contrast, for *C. cosyra* the defended P:C ratio corresponds with the optimum for DEP but is more protein biased than the optima for LEP and lifespan.

There are several possible explanations for this result. The first is that we only measured nutrient regulation for the first 16 days of adult life and this species can live for over one hundred days, so it may be that flies prioritize investing in current reproductive effort early in life, and then later eat a diet that promotes improved survival. However, this strategy of nutrient regulation may be fixed and instead illustrates that in nature sources of nutrients exploited by fruit flies are limited, and only free amino acids can be used as a source for protein (Hendrichs et al., 1993). Therefore, fruit flies may have developed a preference for protein, as shown in our dietary choice experiment, to compensate for the relative scarcity of the resource. Alternatively, there may be strong selection in the field to eat a nutrient blend that maximizes current reproductive success rather than lifespan, because in nature, the presence of adverse conditions and predators means that individuals cannot rely on reaching old age, or realizing their potential lifespan. Instead, selection may favor investing heavily in current fitness if resources are available to reproduce. Investment in lifespan may be adaptive when resources are limiting, and individuals have to survive longer to find them. Thus, in laboratory conditions where food is provided ad libitum, or in nature when food resources are abundant, females may select to prioritize fecundity over lifespan. However, it has been recently reported that *C. cosyra* exhibited an average lifespan of 104 ± 2.8 days when flies were maintained individually in small cages with unrestricted access and the ability to self‐regulate intake of sugar and hydrolyzed yeast in a solid form (unpublished data). This compares well with our results of an average lifespan of approximately 100 days when presenting protein and carbohydrate to *C. cosyra* in liquid form at a ratio of 1:3. In contrast, around a 1:3 ratio, the nutritional landscapes built by Fanson et al. predict an average lifespan between 40 and 50 days in females of the generalist *B. tryoni* (Fanson et al., 2009).

In conclusion, our results indicate that female fecundity is optimized at a low protein intake in *C. cosyra*. This could be attributed to the low requirement for protein by females to produce eggs, which is in stark contrast with generalist species that have been studied where protein is usually the main key nutrient for female reproductive effort. As we did not find evidence for a strong trade‐off between lifespan and reproductive traits in this species, we suggest that experiencing a low‐to‐moderate trade‐off could be a feature of *C. cosyra* to deal with variation in host availability. Nutrients (especially free amino acids) are hard to acquire in nature (Hendrichs et al., 1993), so in the absence of strong lifespan impairment when feeding at a medium P:C ratio (1:3), a nutrient intake that promotes DEP would be advantageous for this species, which depends on host availability. This would maintain the possibility for a short burst of reproductive effort when given the opportunity to do so. Future studies are needed to compare the nutrient intake of a range of host‐specialist species that also investigate how male reproductive effort is affected. Finally, it has been reported that lipid storage could play a role in the lifespan–reproduction trade‐off (Moghadam et al., 2015). Investigating lipid regulation and their effect on life‐history traits using the GF approach might be a next step to understand the link between aging mechanisms and the degree of specialization.

## CONFLICT OF INTEREST

The authors declare no competing interests.

## AUTHORS' CONTRIBUTIONS

CRA, CWW, and SWN conceived the ideas and designed methodology; KM and JH conducted data analysis; KM collected laboratory data; KM led the writing of the manuscript; all authors contributed critically to the drafts and gave final approval for publication.

## DATA AVAILABILITY

Data are available from the Dryad Digital Repository: https://doi.org/10.5061/dryad.146b6.
