# Supplementary material for: Effects of macronutrient intake on the lifespan and fecundity of the marula fruit fly, Ceratitis cosyra (Tephritidae): Extreme lifespan in a host specialist
Source: Ecol Evol. 2017 Oct 22;7(22):9808–17. doi: 10.1002/ece3.3543 (PMC5696426; doi:10.1002/ece3.3543)

**Supplementary information**

**Figure S1: Standard curve to estimate volume from length**

To be able to estimate the volume contained in a pipette tip from the length measured we built a standard curve. We filled pipette tips with a volume ranging from 2 µL to 120 µL and measured the length (mm) at 2 µL intervals. For each step, 13 measures were taken for a total of 736 data points.

**Figure S2: Diagram of cage used to house individual female *C. cosyra* during no-choice experiment.**

**
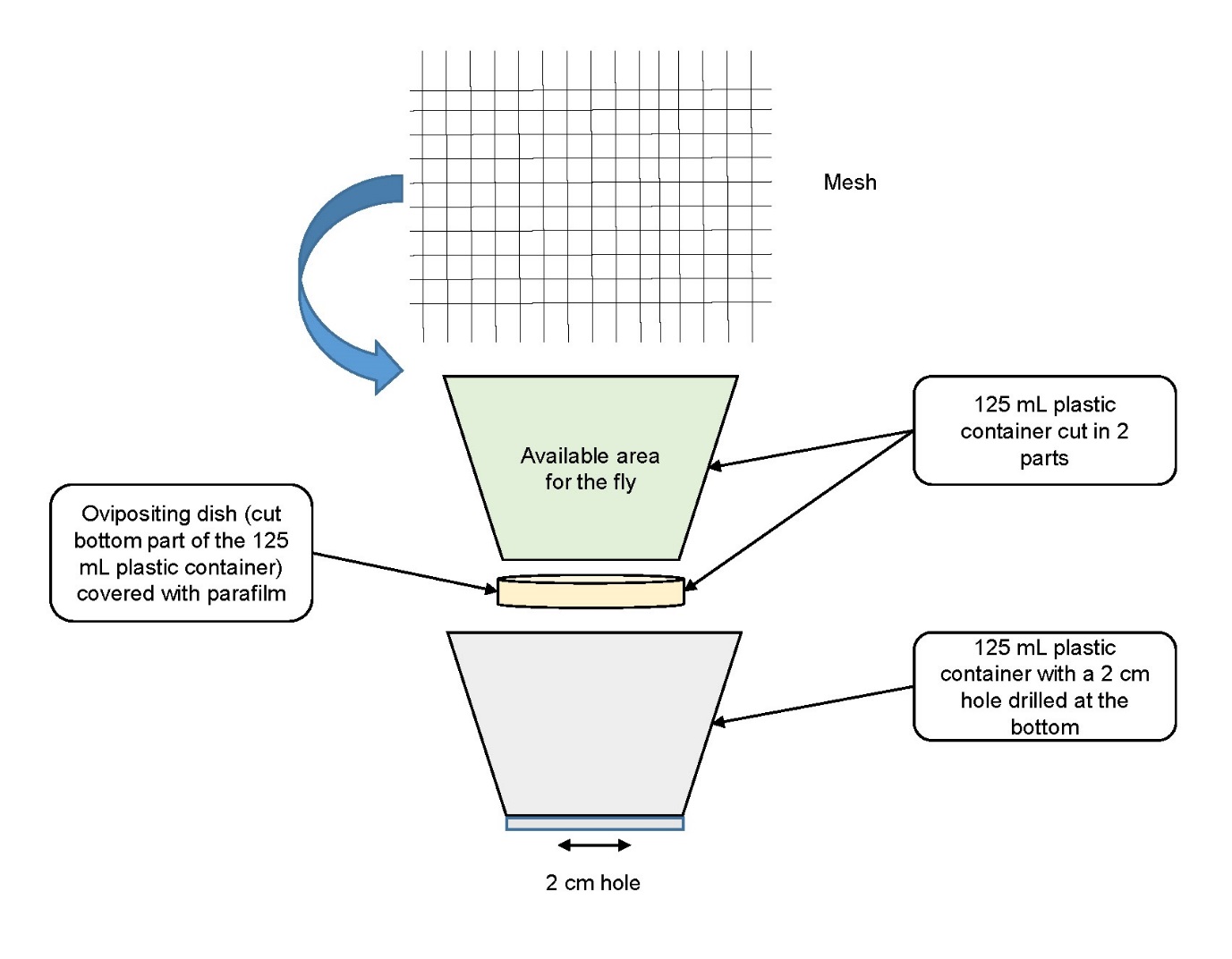
**

In the no-choice experiment, while males were kept in a single 125 mL plastic container, females were kept in an assembly of two 125 mL plastic containers to facilitate the access to the ovipositing dish. A first container was cut at approximatively 1 cm from the bottom. In a second 125 mL plastic container a 2 cm hole was drilled in middle of the floor to easily remove the ovipositing dish. The bottom part of the first container (called ovipositing dish) was filled with 2.5 mL of 10% orange essence (Robertsons, Johannesburg, South Africa) and covered with laboratory film (Parafilm M, Bemis, USA) pierced several times with an entomological pine. The ovipositing dish was then inserted from the top in the second container and then the top part of the first container. This assembly was closed at the top by an insect screen maintained by two rubber bands. Pipette tips were inserted through the insect screen and maintained in a position almost horizontal to avoid dripping.

**Figure S3: Distribution of the 18 liquid diets based on protein and carbohydrates concentration (g/L).**


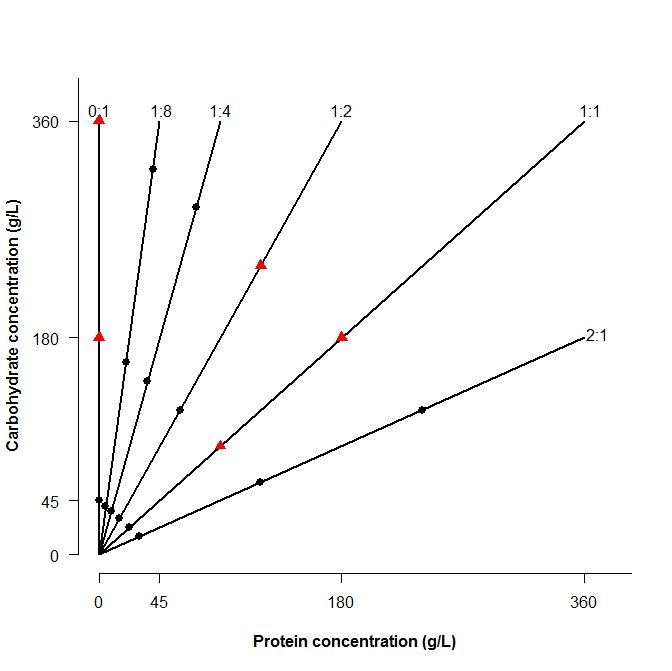


Our 18 liquid diets used in the no-choice experiment are distributed over 6 P:C ratio rails and 3 concentrations (g/L). Each dot or triangle represents one diet. The red rectangles represent the diets that we used in the dietary choice experiment, and correspond to the one also used by Jensen *et al.* (2015). **Pair 1**: 1:1 (180 g/L) vs 0:1 (180 g/L); **Pair 2**: 1:1 (180 g/L) vs 0:1 (360 g/L); **Pair 3**: 1:1 (360 g/L) vs 0:1 (180 g/L); **Pair 4**: 1:1 (360 g/L) vs 0:1 (360 g/L); **Pair 5**: 1:2 (360 g/L) vs 0:1 (360 g/L).

**Figure S4. Average longevity (±SE) for female and male across the different diets. Group sizes are provided in table S3.**

**Figure S5: Female and male nutrient preference under dietary choice**

ns

ns

For both nutrients within each pair we compared the difference between the observed intake and the expected intake to a mean of zero by using a Student t test. Except in pair 3, nutrient intake differed from a mean of zero for both sexes. **Female:** **Pair 1:** P (t = 2.52, p.value < 0.05); C (t = -2.52, p.value < 0.05); **Pair 2:** P (t = 4.28, p.value < 0.001); C (t = -4.28, p.value < 0.001); **Pair 3:** P (t = 0.82, p.value > 0.05); C (t = NA, p.value = NA); **Pair 4:** P (t = 5.43, p.value < 0.001); C (t = -5.43, p.value < 0.001); **Pair 5:** P (t = 2.7, p.value < 0.05); C (t = -2.7, p.value < 0.05). **Male:** **Pair 1:** P (t = 2.97, p.value < 0.01); C (t = -2.97, p.value < 0.01); **Pair 2:** P (t = 8.16, p.value < 0.001); C (t = -8.16, p.value < 0.001); **Pair 3:** P (t = 0.62, p.value > 0.05); C (t = NA, p.value = NA); **Pair 4:** P (t = 4.87, p.value < 0.001); C (t = -4.87, p.value < 0.001); **Pair 5:** P (t = 3.62, p.value < 0.05); C (t = -3.62, p.value < 0.05).

**Figure S6: Female and male diet preference under dietary choice**

ns

ns

**Pair 1**: 1:1 (180 g/L) vs 0:1 (180 g/L); **Pair 2**: 1:1 (180 g/L) vs 0:1 (360 g/L); **Pair 3**: 1:1 (360 g/L) vs 0:1 (180 g/L); **Pair 4**: 1:1 (360 g/L) vs 0:1 (360 g/L); **Pair 5**: 1:2 (360 g/L) vs 0:1 (360 g/L).

As the data did not follow a normal distribution and it was not possible to obtain a normal distribution by transforming the data, we used a Wilcoxon Signed Rank test to compare consumption across diet pairs. In both sexes, except in pair 3, a significant preference for the diet containing protein was detected, irrespective of nutrient content. **Female**: **Pair 1**: V = 112, p.value < 0.05; **Pair 2**: V = 126, p.value < 0.01; **Pair 3**: V = 84, p.value > 0.05; **Pair 4**: V = 117, p.value < 0.001; **Pair 5**: V = 111, p.value < 0.05. **Male**: **Pair 1**: V = 117, p.value < 0.01; **Pair 2**: V = 135, p.value < 0.001; **Pair 3**: V = 81, p.value > 0.05; **Pair 4**: V = 127, p.value < 0.001; **Pair 5**: V = 123, p.value < 0.01.

**Figure S7 Average total intake (±SE) of P and C in replicates 1 and 2 when females are given the choice between two diets over a 16 days feeding period. The blue circles represent the first replicate and the red triangles the second replicate. The large red triangle and blue circle are the regulated intake points.**


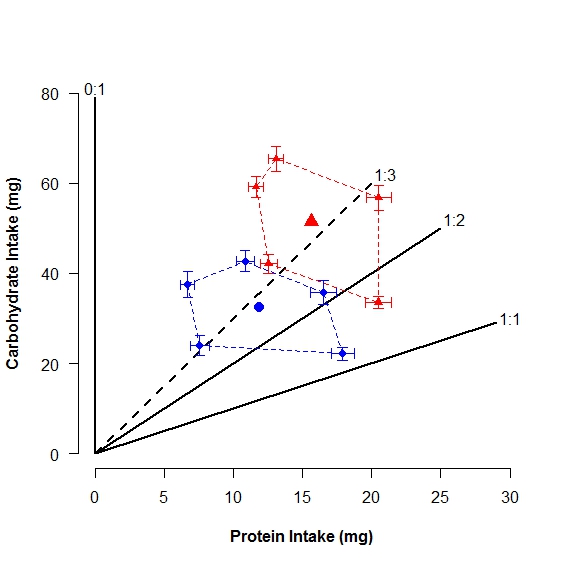


**Figure S8. Average total intake (±SE) of P and C in replicates 1 and 2 when males are given the choice between two diets over a 16 days feeding period. The blue circles represent the first replicate and the red triangles the second replicate. The large red triangle and blue circle are the regulated intake points.**


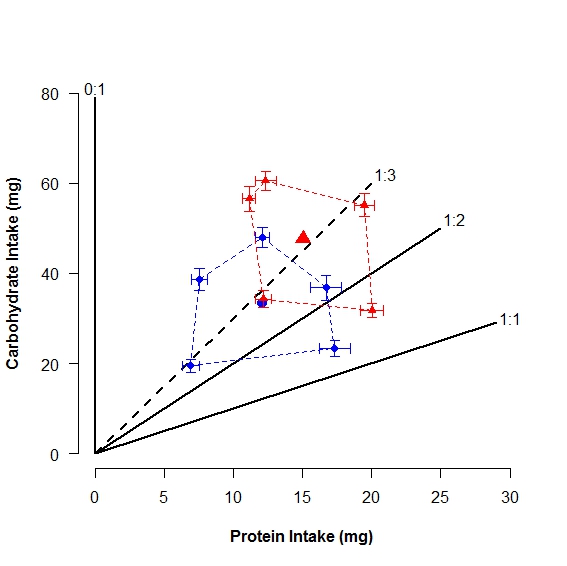

Supplement: Supplementary file 1 [file ECE3-7-9808-s001.docx]
