# Supplementary material for: Effects of macronutrient intake on the lifespan and fecundity of the marula fruit fly, Ceratitis cosyra (Tephritidae): Extreme lifespan in a host specialist
Source: Ecol Evol. 2017 Oct 22;7(22):9808–17. doi: 10.1002/ece3.3543 (PMC5696426; doi:10.1002/ece3.3543)
Supplement: Supplementary file 2 [file ECE3-7-9808-s002.docx]

**Table S1: Experimental diets fed to female and male *C. cosyra* in either no-choice or choice experiment.**

| P:C | Concentration (g/L) | Sucrose (g/L) | Amino Acids (g/L) | Vitamin (g/L) | Cholesterol (g/L) | RNA (g/L) | Wesson salt (g/L) |
| --- | --- | --- | --- | --- | --- | --- | --- |
| 0:1 | 360 | 360.00 | 0.00 | 3.6 | 4.0 | 10.0 | 10.0 |
|  | 180 | 180.00 | 0.00 | 3.6 | 4.0 | 10.0 | 10.0 |
|  | 45 | 45.00 | 0.00 | 3.6 | 4.0 | 10.0 | 10.0 |
| 1:8 | 360 | 320.00 | 40.00 | 3.6 | 4.0 | 10.0 | 10.0 |
|  | 180 | 160.00 | 20.00 | 3.6 | 4.0 | 10.0 | 10.0 |
|  | 45 | 40.00 | 5.00 | 3.6 | 4.0 | 10.0 | 10.0 |
| 1:4 | 360 | 288.00 | 72.00 | 3.6 | 4.0 | 10.0 | 10.0 |
|  | 180 | 144.00 | 36.00 | 3.6 | 4.0 | 10.0 | 10.0 |
|  | 45 | 36.00 | 9.00 | 3.6 | 4.0 | 10.0 | 10.0 |
| 1:2 | 360 | 240.00 | 120.00 | 3.6 | 4.0 | 10.0 | 10.0 |
|  | 180 | 120.00 | 60.00 | 3.6 | 4.0 | 10.0 | 10.0 |
|  | 45 | 30.00 | 15.00 | 3.6 | 4.0 | 10.0 | 10.0 |
| 1:1 | 360 | 180.00 | 180.00 | 3.6 | 4.0 | 10.0 | 10.0 |
|  | 180 | 90.00 | 90.00 | 3.6 | 4.0 | 10.0 | 10.0 |
|  | 45 | 22.50 | 22.50 | 3.6 | 4.0 | 10.0 | 10.0 |
| 2:1 | 360 | 120.00 | 240.00 | 3.6 | 4.0 | 10.0 | 10.0 |
|  | 180 | 60.00 | 120.00 | 3.6 | 4.0 | 10.0 | 10.0 |
|  | 45 | 15.00 | 30.00 | 3.6 | 4.0 | 10.0 | 10.0 |

All dry ingredients were dissolved in filtered water (50°C) and vigorously combined with a vortex mixer (Agimatic-N, J.P Selecta, Spain). Finally, 0.13% (v/v) of blue food dye (1.3 mL/L) to facilitate measurement of diet consumption, and Nipagin (1.5 g/L) to prevent microbial infection were added.

Experimental diets were provided to individual flies in yellow plastic 200 µL pipette tips (ROLL s.a.s, Italy).
