# Supplementary material for: Effects of macronutrient intake on the lifespan and fecundity of the marula fruit fly, Ceratitis cosyra (Tephritidae): Extreme lifespan in a host specialist
Source: Ecol Evol. 2017 Oct 22;7(22):9808–17. doi: 10.1002/ece3.3543 (PMC5696426; doi:10.1002/ece3.3543)
Supplement: Supplementary file 3 [file ECE3-7-9808-s003.docx]

**Table S2: Mixture of micronutrients incorporated into all experimental diets fed to *C. cosyra***

|  |  | g/(100g) | Producer (Product #) |
| --- | --- | --- | --- |
| Amino acid mixture | l-Alanine | 4.87 | Merck (1.01007.0100) |
|  | l-Arginine | 6.67 | Sigma (11039) |
|  | l-Aspartic acid | 7.05 | Merck (1.00126.0100) |
|  | l-Cysteine | 2.55 | Merck (1.02839.0100) |
|  | l-Glutamic acid | 24.51 | Merck (8.14632.0250) |
|  | Glycine | 5.62 | Merck (1.04201.0100) |
|  | l-Histidine | 2.85 | Merck (1.04351.0100) |
|  | l-Isoleucine | 3.52 | Merck (1.05362.0100) |
|  | l-Leucine | 6.75 | Merck (1.05360.0250) |
|  | l-Lysine monohydrochloride | 3.67 | Merck (1.05700.0100) |
|  | l-Methionine | 1.72 | Merck (1.05707.0025) |
|  | l-Phenylalanine | 4.42 | Merck (1.07256.0100) |
|  | l-Proline | 7.80 | Merck (5370-GM) |
|  | l-Serine | 4.87 | Merck (1.07769.0100) |
|  | l-Threonine | 3.37 | Merck (1.08411.0100) |
|  | l-Tryptophan | 1.80 | Merck (1.08374.0010) |
|  | l-Tyrosine | 3.00 | Merck (1.08371.0025) |
|  | l-Valine | 4.95 | Merck (1.08495.0100) |
| Cholesterol |  | 100 | Sigma (C3045) |
| RNA |  | 100 | Sigma (R6625) |
| Sucrose |  | 100 | Merck (573113) |
| Vanderzant vitamin mixture | Ascorbic acid | 27.00 | Sigma (V1007) |
|  | Biotin | 0.002 |  |
|  | Choline chloride | 5.00 |  |
|  | Folic acid | 0.03 |  |
|  | Glucose | 64.70 |  |
|  | Inositol | 2.00 |  |
|  | Niacinamide | 0.10 |  |
|  | Pantothenic acid hemicalcium | 0.10 |  |
|  | Pyridoxine HCl | 0.03 |  |
|  | Riboflavin | 0.05 |  |
|  | Thiamin HCl | 0.03 |  |
|  | Tocopherol type VI | 0.80 |  |
|  | Vitamin B12 | 0.20 |  |
| Wesson salt mixture | Calcium carbonate | 21.00 | Sigma |
|  | Copper sulfate | 0.04 |  |
|  | Ferric phosphate | 1.47 |  |
|  | Magnesium sulfate | 9.00 |  |
|  | Manganese sulfate | 0.02 |  |
|  | Potassium aluminium sulfate | 0.01 |  |
|  | Potassium chloride | 12.00 |  |
|  | Potassium iodide | 0.005 |  |
|  | Potassium phosphate monobasic | 31.00 |  |
|  | Sodium chloride | 10.50 |  |
|  | Sodium fluoride | 0.06 |  |
|  | Tricalcium phosphate | 14.90 |  |
| Nipagin |  | 1.5 | Sigma (H3647) |
