# Supplementary material for: Effects of macronutrient intake on the lifespan and fecundity of the marula fruit fly, Ceratitis cosyra (Tephritidae): Extreme lifespan in a host specialist
Source: Ecol Evol. 2017 Oct 22;7(22):9808–17. doi: 10.1002/ece3.3543 (PMC5696426; doi:10.1002/ece3.3543)
Supplement: Supplementary file 4 [file ECE3-7-9808-s004.docx]

**Table S3: Group size for the data analysis in the no-choice experiment. Initial group size at the beginning of the experiment is given in parentheses.**

| **P:C** | **Concentration (g/L)** | **Female** | **Male** |
| --- | --- | --- | --- |
| **0:1** | **360** | 5 (10) | 9 (10) |
|  | **180** | 8 (10) | 9 (10) |
|  | **45** | 9 (10) | 9 (10) |
| **1:8** | **360** | 8 (10) | 9 (10) |
|  | **180** | 6 (10) | 10 |
|  | **45** | 10 | 9 (10) |
| **1:4** | **360** | 6 (7) | 6 (7) |
|  | **180** | 6 (7) | 6 (7) |
|  | **45** | 7 | 6 (7) |
| **1:2** | **360** | 6 (7) | 5 (7) |
|  | **180** | 5 (7) | 7 |
|  | **45** | 6 (7) | 7 |
| **1:1** | **360** | 5 (7) | 7 |
|  | **180** | 6 (7) | 6 (7) |
|  | **45** | 5 (7) | 7 |
| **2:1** | **360** | 7 | 6 (7) |
|  | **180** | 7 | 7 |
|  | **45** | 7 | 7 |
