# Supplementary material for: Effects of macronutrient intake on the lifespan and fecundity of the marula fruit fly, Ceratitis cosyra (Tephritidae): Extreme lifespan in a host specialist
Source: Ecol Evol. 2017 Oct 22;7(22):9808–17. doi: 10.1002/ece3.3543 (PMC5696426; doi:10.1002/ece3.3543)
Supplement: Supplementary file 5 [file ECE3-7-9808-s005.docx]

**Table S4. Difference in average loss (± SE) of liquid diet per day in evaporation controls between replicates.**

| **Diet** | **Average loss replicate 1 / day (µL)** | **Average loss replicate 2 / day (µL)** |
| --- | --- | --- |
| 1-1(180) | 18.40 ± 2.08 | 8.44 ± 1.05 |
| 0-1(360) | 17.63 ± 1.16 | 11.52 ± 1.89 |
| 0-1(180) | 22.67 ± 1.66 | 14.93 ± 2.46 |
| 1-1(360) | 6.56 ± 0.92 | 3.99 ± 0.73 |
| 1-2(360) | 10.66 ± 1.18 | 7.69 ± 0.96 |
